# The association between Mycoplasma pneumoniae infection and speech and language impairment: A nationwide population-based study in Taiwan

**DOI:** 10.1371/journal.pone.0180402

**Published:** 2017-07-03

**Authors:** Ching-Shu Tsai, Vincent Chin-Hung Chen, Yao-Hsu Yang, Tai-Hsin Hung, Mong-Liang Lu, Kuo-You Huang, Michael Gossop

**Affiliations:** 1 Department of Psychiatry, Chang Gung Memorial Hospital and University, Chiayi, Taiwan; 2 Graduate Institute of Clinical Medical Sciences, Chang Gung University, Taoyuan, Taiwan; 3 Chang Gung Institute of Technology, Taoyuan, Taiwan; 4 Department for Traditional Chinese Medicine, Chang Gung Memorial Hospital, Chiayi, Taiwan; 5 Center of Excellence for Chang Gung Research Datalink, Chang Gung Memorial Hospital, Chiayi, Taiwan; 6 Institute of Occupational Medicine and Industrial Hygiene, National Taiwan University College of Public Health, Taipei, Taiwan; 7 School of Traditional Chinese Medicine, College of Medicine, Chang Gung University, Taoyuan, Taiwan; 8 Department of Psychiatry, Wan-Fang Hospital and School of Medicine, College of Medicine, Taipei Medical University, Taipei, Taiwan; 9 Department of Speech Language Pathology and Audiology, Chung Shan Medical University and Hospital, Taichung, Taiwan; 10 National Addiction Centre, King’s College, London, United Kingdom; Waseda University, JAPAN

## Abstract

Manifestations of *Mycoplasma pneumoniae* infection can range from self-limiting upper respiratory symptoms to various neurological complications, including speech and language impairment. But an association between *Mycoplasma pneumoniae* infection and speech and language impairment has not been sufficiently explored. In this study, we aim to investigate the association between *Mycoplasma pneumoniae* infection and subsequent speech and language impairment in a nationwide population-based sample using Taiwan’s National Health Insurance Research Database. We identified 5,406 children with *Mycoplasma pneumoniae* infection (International Classification of Disease, Revision 9, Clinical Modification code 4830) and compared to 21,624 age-, sex-, urban- and income-matched controls on subsequent speech and language impairment. The mean follow-up interval for all subjects was 6.44 years (standard deviation = 2.42 years); the mean latency period between the initial *Mycoplasma pneumoniae* infection and presence of speech and language impairment was 1.96 years (standard deviation = 1.64 years). The results showed that *Mycoplasma pneumoniae* infection was significantly associated with greater incidence of speech and language impairment [hazard ratio (HR) = 1.49, 95% CI: 1.23–1.80]. In addition, significantly increased hazard ratio of subsequent speech and language impairment in the groups younger than 6 years old and no significant difference in the groups over the age of 6 years were found (HR = 1.43, 95% CI:1.09–1.88 for age 0–3 years group; HR = 1.67, 95% CI: 1.25–2.23 for age 4–5 years group; HR = 1.14, 95% CI: 0.54–2.39 for age 6–7 years group; and HR = 0.83, 95% CI:0.23–2.92 for age 8–18 years group). In conclusion, *Mycoplasma pneumoniae* infection is temporally associated with incident speech and language impairment.

## Introduction

*Mycoplasma pneumoniae (M*. *pneumoniae)*, belonging to the class Mollicutes, is a ubiquitous and well-established pathogen of the respiratory tract. Manifestations of *M*. *pneumoniae* infection can range from self-limiting upper respiratory illness to severe pneumonia. Between 10% and 20% of radiologically proven pneumonia cases in endemic periods and up to 50% of all cases in epidemic periods are caused by *M*. *pneumoniae* [1, 2].

About 25% of *M*. *pneumoniae* infected persons may be complicated by the involvements of various extrapulmonary systems, and the central nervous system (CNS) is the most frequently affected [3, 4]. Within hospitalized patients with serologically confirmed *M*. *pneumoniae* infection, approximately 1–10% is associated with neurological manifestations [5]. Cerebellar syndrome, polyradiculitis, cranial nerve palsies, aseptic meningitis, encephalitis, meningoencephalitis, acute disseminated encephalomyelitis, optic neuritis, diplopia, cranial nerve palsy, ataxia, choreoathetosis, and ascending paralysis (Guillain-Barre syndrome) are examples of neurological complications in *M*. *pneumoniae* infection [6]. More than 80% of patients with CNS findings have prior or concomitant respiratory illness [7]. The time interval between the onset of respiratory symptoms and neurological symptoms varies from 2 to 14 days [8, 9]. But late onset encephalitis after several days to a few weeks was also reported [5].

*M*. *pneumoniae* infections can occur in children of all ages. Many *M*. *pneumoniae* infections are subclinical, a finding that is particularly common in children younger than 5 years of age [10]. But age-specific attack rates for *M*. *pneumoniae* pneumonia could be observed. The rate among children aged five to nine years was about two times for younger children and four times for adults [11]. Within hospitalized pediatric patients with *M*. *pneumoniae* infection, 25% have neurological symptoms [12]. And 5–10% of childhood encephalitis is attributed to *M*. *pneumoniae* [13]. Although there was a self-limited course and favorable outcome after an acute *M*. *pneumoniae* infection, significant neurological sequelae in 45% of hospitalized pediatric patients have been reported [12].

There were only two case reports of subsequent speech and language impairment after *M*. *pneumoniae* infection in our literature review [14, 15]. To our knowledge, there is no large scale study to explore the association between *M*. *pneumoniae* infection and speech and language impairment. This study investigates the association between *M*. *pneumoniae* infection and subsequent speech and language impairment in a nationwide population-based sample.

## Materials and methods

### Database

Data used were obtained from the Taiwan National Health Insurance Research Database (NHIRD). The NHIRD was launched on March 1, 1995 and is maintained by the Department of Health and the National Health Research Institutes (NHRI). It covers more than ninety-nine per cent of the national population of Taiwan [16] and provides comprehensive patient data, including demographic data, dates and numbers of clinical visits, dates of admission and discharge, the names of prescribed drugs, dosages, and prescription duration, operations, examinations, diagnostic codes in the format of the International Classification of Disease, Revision 9, Clinical Modification (ICD-9-CM), and physician specialties: these provide valuable information for epidemiological study [17]. The NHRI has provided these data to scientists for research purposes since 2000. In concert with the Bureau of the National Health Insurance, the NHRI extracted a randomly sampled representative database of 1,000,000 people from the registry of all enrolees via a systemic sampling method to form the Longitudinal Health Insurance Database in 2005 (LHID 2005). The LHID contained all reimbursement claims records from 1996 to 2013. There are no statistically significant differences in age, sex, or health care costs between this sample and all enrollees [18].

### Study subjects and design

For the difficult incubation and limited application of the polymerase chain reaction (PCR) techniques for *M*. *pneumoniae* in the clinical settings, the serological diagnosis is widely used. The sensitivity of the serological diagnosis is similar to that of the PCR methods [19]. In Taiwan, *M*. *pneumoniae* infection is diagnosed by clinical evaluation and serologic test results [20], including specific IgM present or a greater than 4-fold increase in specific IgG levels in a blood sample [21].

**Fig 1:**
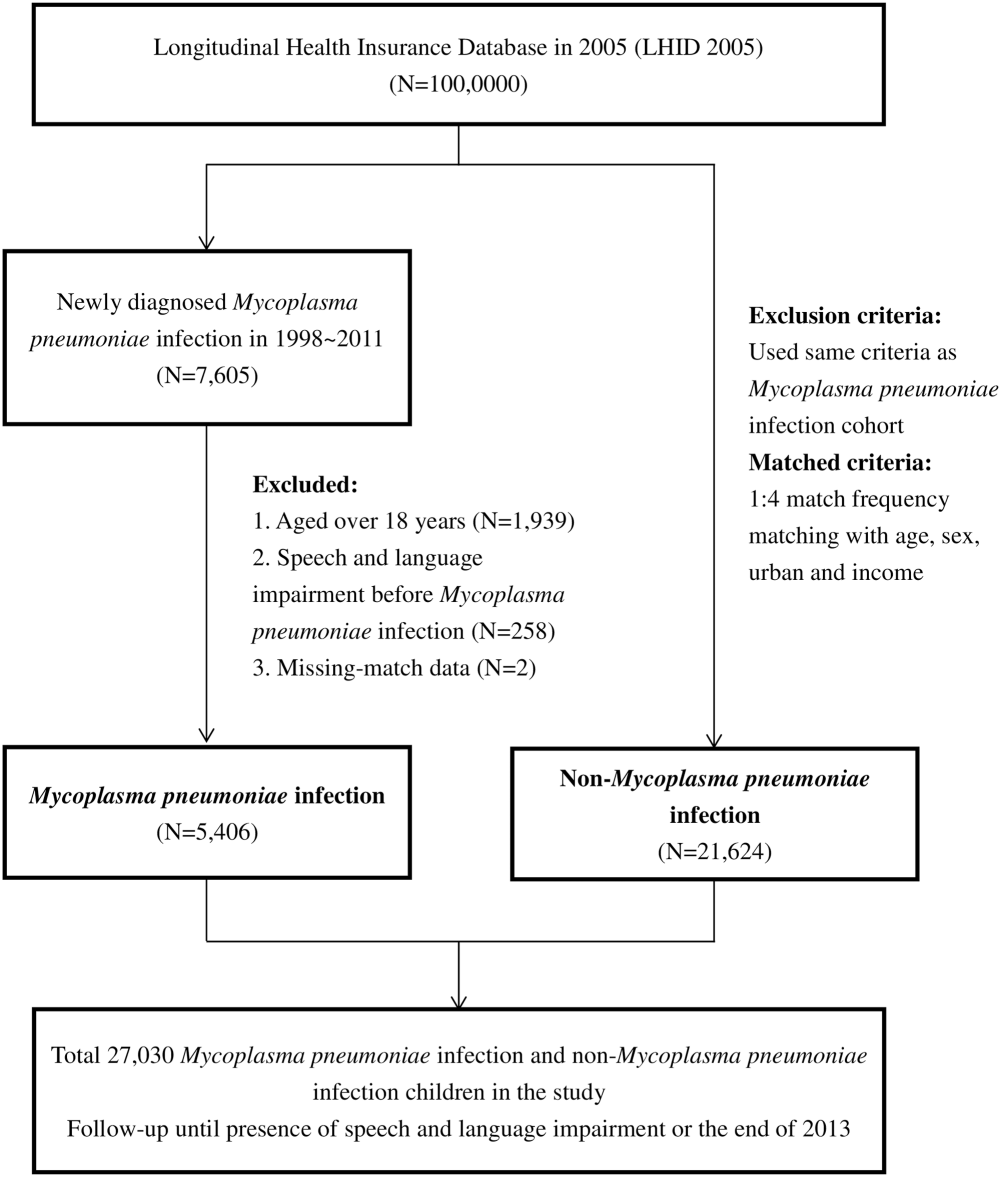
Flow chart of data collection.

The study subjects were comprised of insured children under 18 years of age between 1998 and 2011. Inclusion criteria for the *M*. *pneumoniae* infection group included at least two ambulatory claims within one year or at least one inpatient claim with a diagnosis of *M*. *pneumoniae* infection (ICD-9-CM code 4830). The foregoing definition is consistent with previous research using this database [22]. Children who have diagnostic codes of developmental speech or language disorder (ICD-9-CM codes 315.3, 315.31–39), aphasia (ICD-9-CM code 784.3), voice and resonance disorders (ICD-9-CM codes 784.4, 784.40–49), and other speech disturbance (ICD-9-CM codes 784.5, 784.51–59) or received speech therapy before *M*. *pneumoniae* infection were excluded from the analysis. A total of 5,406 children with *M*. *pneumoniae* infection were eligible. The children matching on age, sex, urban and income from the remaining subjects as comparison group were randomly sampled at a 1:4 ratio. The process is shown in Fig 1.

### Case identification of speech and language impairment

Adult onset fluency disorder (ICD-9-CM code 307.0), developmental speech or language disorder (ICD-9-CM codes 315.3, 315.31–39), aphasia (ICD-9-CM code 784.3), voice and resonance disorders (ICD-9-CM codes 784.4, 784.40–49), and other speech disturbance (ICD-9-CM codes 784.5, 784.51–59) were operationalized as speech and language impairment. Cases that had at least two ambulatory claims within one year or at least one inpatient claim with these diagnostic codes were identified and collected for analysis. In addition, cases of speech therapy without above-mentioned diagnostic codes were also classified as the group of speech and language impairment.

### Confounding factors

The analyses were adjusted for potential confounders which had been reported in relation to speech and language impairment in previous studies, including preterm labor and small for gestational age (ICD-9-CM codes 765–765.19) [23, 24], perinatal complications (ICD-9-CM codes 760–764, 766–779 and V137) [24], autistic spectrum disorder (ICD-9-CM codes 299) [25, 26], intellectual disability (ICD-9-CM codes 317, 318, 319) [26, 27], other disorders of the CNS (ICD-9-CM codes 340–349) including cerebral palsy (ICD-9-CM codes 343) [28], attention deficit hyperactivity disorder (ICD-9-CM codes 314) [29], otitis media (ICD-9-CM codes 381.0–381.4 and 382) [30, 31], hearing loss (ICD-9-CM codes 389) [24, 26], and traumatic brain injury (ICD-9-CM codes 800–804 and 850–854) [32].

### Statistical analysis

The distribution of demographic factors and comorbidities were compared between the *M*. *pneumoniae* infection group and the comparison group. We used the Kaplan–Meier methods to estimate cumulative incidences of speech and language impairment and the log-rank tests to examine the differences. Finally, Cox proportional hazards models were used to compute the hazard ratios accompanying 95% confidence intervals (CIs) after adjustment for age, sex, urban, income, and other confounders. Two-tailed p value = 0.05 was considered significant. Individuals who were deceased during the study period and/or were from the beneficiaries register lost to follow-up were omitted from the analysis. All analyses were conducted using SAS statistical software (Version 9.4; SAS Institute, Cary, NC, USA).

### Ethics statement

The study was approved by the Institutional Review Board of Chang Gung Memorial Hospital. Written informed consent was exempted.

## Results

### Characteristics of subjects

The study sample comprised 5,406 individuals with *M*. *pneumoniae* infection and 21,624 age-, sex-, urban- and income-matched individuals ascertained from the database. The characteristics are described and compared in Table 1. Compared to the comparison group, individuals with *M*. *pneumoniae* infection had more perinatal complications, other disorders of the CNS, otitis media, traumatic brain injury and subsequent speech and language impairment, and less autistic spectrum disorder. The mean follow-up interval for all subjects was 6.44 years (standard deviation = 2.42 years). Mean latency period between the initial *M*. *pneumoniae* infection and presence of speech and language impairment was 1.96 years (standard deviation = 1.64 years).

**Table 1:** Characteristics of *M*. *pneumoniae* infection cases and their matched controls.

Characteristic	MP^1^ infection		Non-MP^1^ infection		P value^2^
	N	%	N	%	
**Sex**					
Girl	2719	50.30	10876	50.30	>0.9999
Boy	2687	49.70	10748	49.70	
**Age at entry (years)**					
0–3	967	17.89	3868	17.89	>0.9999
4–5	1734	32.08	6936	32.08	
6–7	1198	22.16	4792	22.16	
8–18	1507	27.88	6028	27.88	
**Preterm labor and small for gestational age**					
No	5267	97.43	21115	97.65	0.3501
Yes	139	2.57	509	2.35	
**Perinatal complications**					
No	4683	86.63	19322	89.35	<0.0001
Yes	723	13.37	2302	10.65	
**Autistic spectrum disorder**					
No	5398	99.85	21546	99.64	0.0130
Yes	8	0.15	78	0.36	
**Intellectual disability**					
No	5388	99.67	21531	99.57	0.3180
Yes	18	0.33	93	0.43	
**Other disorders of the CNS**					
No	5223	96.61	21130	97.72	<0.01
Yes	183	3.39	494	2.28	
**Attention deficit hyperactivity disorder**					
No	5313	98.28	21250	98.27	0.96
Yes	93	1.72	374	1.73	
**Otitis media**					
No	3167	58.58	15403	71.23	<0.01
Yes	2239	41.42	6221	28.77	
**Hearing loss**					
No	5361	99.17	21415	99.03	0.36
Yes	45	0.83	209	0.97	
**Traumatic brain injury**					
No	5212	96.41	21134	97.73	<0.01
Yes	194	3.59	490	2.27	
**Speech and language impairment**					
No	5254	97.19	21220	98.13	<0.01
Yes	152	2.81	404	1.87	

^**1**^MP = *M*. *pneumoniae***;**

^2^Chi-square test

### Association between potential risk factors and risk of speech and language impairment

As shown in Table 2, the hazard ratio of speech and language impairment in boys was higher than the ratio in girls (hazard ratio = 2.00, 95% CI: 1.67–2.39). Compared to the children aged eight to eighteen years, children aged from zero to three years have the highest risk (hazard ratio = 24.25, 95% CI: 14.78–39.78), followed by the children aged 4–5 years (hazard ratio = 11.19, 95% CI: 6.82–18.37) and the children aged 6–7 years (hazard ratio = 2.98, 95% CI: 1.69–5.24). There were significant increased hazard ratios among the prior history of preterm labor and small for gestational age (hazard ratio = 2.05, 95% CI: 1.35–3.13), intellectual disability (hazard ratio = 4.26, 95% CI: 1.91–9.51), or other disorders of the CNS (hazard ratio = 1.97, 95% CI: 1.34–2.89).

**Table 2:** Hazard ratios of factors associated with speech and language impairment via Cox regression analysis.

Variables	Unadjusted hazard ratio			Adjusted hazard ratio		
	Hazard ratio	95%CI	P value	Hazard ratio	95%CI	P value
**MP infection**						
Non-MP(reference)	1.00			1.00		
MP	1.52	1.26–1.83	<.0001	1.49	1.23–1.8	<.0001
**Sex**						
Girl(reference)	1.00			1.00		
Boy	2.12	1.78–2.53	<.0001	2.00	1.67–2.39	<.0001
**Age at entry (years)**						
0–3	22.93	14.05–37.43	<.0001	24.25	14.78–39.78	<.0001
4–5	10.79	6.59–17.67	<.0001	11.19	6.82–18.37	<.0001
6–7	2.98	1.69–5.25	0.0002	2.98	1.69–5.24	0.0002
8-18(reference)	1.00			1.00		
**Preterm labor and small for gestational age**						
No(reference)	1.00			1.00		
Yes	2.53	1.77–3.62	<.0001	2.05	1.35–3.12	0.0008
**Perinatal complications**						
No(reference)	1.00			1.00		
Yes	1.44	1.14–1.81	0.0021	0.96	0.73–1.26	0.7675
**Autistic spectrum disorder**						
No(reference)	1.00			1.00		
Yes	1.19	0.30–4.78	0.8032	0.53	0.12–2.3	0.3937
**Intellectual disability**						
No(reference)	1.00			1.00		
Yes	3.32	1.58–6.98	0.0016	4.33	1.94–9.66	0.0004
**Other disorders of the CNS**						
No(reference)	1.00			1.00		
Yes	2.45	1.72–3.51	<.0001	1.98	1.35–2.9	0.0005
**Attention deficit hyperactivity disorder**						
No(reference)	1.00			1.00		
Yes	1.13	0.60–2.11	0.71	1.84	0.95–3.56	0.0701
**Otitis media**						
No(reference)	1.00			1.00		
Yes	1.01	0.84–1.21	0.9091	1.11	0.92–1.33	0.2813
**Hearing loss**						
No(reference)	1.00			1.00		
Yes	1.82	0.94–3.52	0.0748	1.65	0.84–3.25	0.1439
**Traumatic brain injury**						
No(reference)	1.00			1.00		
Yes	0.64	0.33–1.23	0.1789	0.83	0.43–1.62	0.5906

MP = *M*. *pneumoniae*

### Association between *M*. *pneumoniae* infection and risk of speech and language impairment

**Fig 2:**
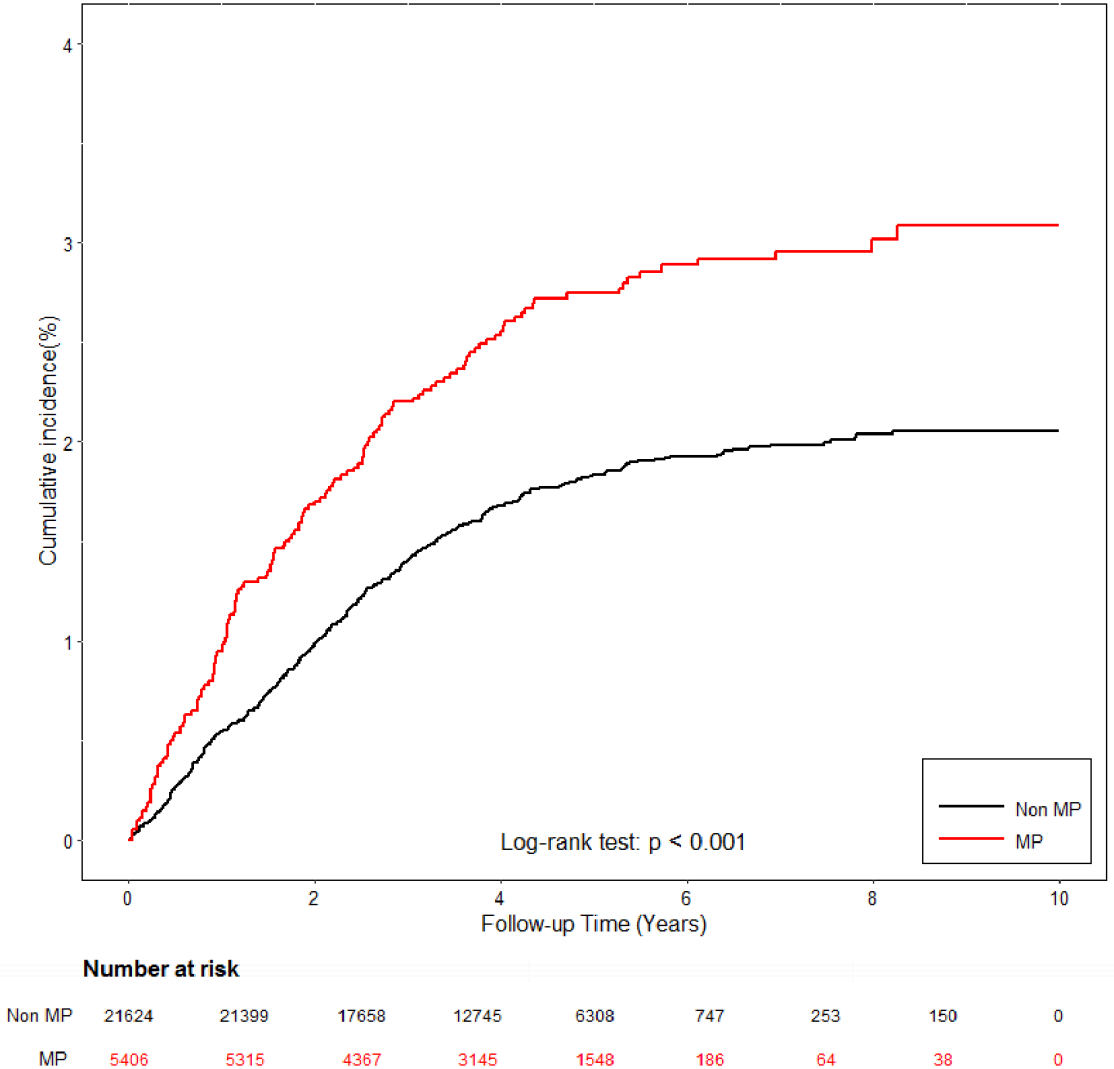
Cumulative incidence of speech and language impairment in cohorts with/without *Mycoplasma pneumoniae* infection.

Analyses of associations of interest are summarized in Table 2 and shown in Fig 2. In the fully adjusted Cox regression model, *M*. *pneumoniae* infection was associated with a greater incidence of speech and language impairment (hazard ratio = 1.49, 95% CI: 1.23–1.80) after adjusting for age, sex, urban, income, preterm labor and small for gestational age, perinatal complications, autistic spectrum disorder, intellectual disability, other disorders of the CNS, attention deficit hyperactivity disorder, otitis media, hearing loss and traumatic brain injury.

### Association between the age of *M*. *pneumoniae* infection and risk of speech and language impairment

The present study also showed that the hazard ratios of subsequent speech and language impairment were modified in different ages of *M*. *pneumoniae* infection. Compared with the same age groups without *M*. *pneumoniae* infection, the hazard ratio of subsequent speech and language impairment in the groups younger than 6 years old was significantly increased, and there was no significant difference in the groups over the age of 6 years. The hazard ratio of *M*. *pneumoniae* infection in the age 0–3 years group, age 4–5 years group, age 6–7 years group and age 8–18 years group was 1.43 (95% CI:1.09–1.88), 1.67 (95% CI: 1.25–2.23), 1.14 (95% CI: 0.54–2.39) and 0.83 (95% CI:0.23–2.92), respectively (Table 3).

**Table 3:** Hazard ratios for speech and language impairment in subgroups of different age groups of *M*. *pneumoniae* infection.

Variables	Unadjusted hazard ratio			Adjusted hazard ratio		
	Hazard ratio	95%CI	P value	Hazard ratio	95%CI	P value
**Age of MP infection (years)**						
0–3	1.51	1.16–1.98	0.0024	1.43	1.09–1.88	0.0109
4–5	1.68	1.26–2.23	0.0004	1.67	1.25–2.23	0.0005
6–7	1.13	0.54–2.36	0.7551	1.14	0.54–2.39	0.739
8–18	0.86	0.25–2.98	0.8074	0.83	0.23–2.92	0.769

MP = *M*. *pneumoniae*

## Discussion

To our knowledge, this is the first study to investigate the relationship between *M*. *pneumoniae* infection and speech and language impairment using a nationwide longitudinal dataset. The results identified a greater risk for speech and language impairment with *M*. *pneumoniae* infection, after adjusting for age, sex, urban, income, preterm labor and small for gestational age, perinatal complications, autistic spectrum disorder, intellectual disability, other disorders of the CNS, attention deficit hyperactivity disorder, otitis media, hearing loss and traumatic brain injury. In the present study, the mean latency period from initial *M*. *pneumoniae* infection to subsequent speech and language impairment was 1.96 years.

To our knowledge, there were only two case reports about speech and language impairment after *M*. *pneumoniae* infection [14, 15]. One reported two female children of 5 and 8 years of age with an apparent *M*. *pneumoniae* infection, and both showed symptoms of cerebellar syndrome, including ataxia, hypotonia, dysarthric speech and dysmetria initially. An initial brain MRI in one girl of 5-year-old was normal, but evident signs of global cerebellar atrophy in follow-up brain MRI performed at 6 and 24 months were present. Incomplete resolution of cerebellar dysfunction with speech problem and fine motor impairment was found three years later. The other girl of 8-year-old displayed striking cerebellar swelling in initial brain MRI. The follow-up brain MRI performed at two months showed normal signal in the cerebellum, but a loss of the volume of both cerebellum hemispheres with preservation of vermis. She was asymptomatic one year after the event [14]. Another case report was 5-year-old boy with a confirmed *M*. *pneumoniae* infection. He was admitted due to right lower lobe pneumonia with pleural effusion. But prolonged generalized tonic or tonic-clonic convulsion on the seventh day was observed. Brain imaging showed bilateral thalamic lesions without striatal or white-matter involvement. Even almost complete neurological recovery by 2 months, significant dysarthria was still present and persisted at 9 months [15]. Results from these case reports indicated that *M*. *pneumoniae* infection was associated with speech and language impairment, but the presence or not of such neurological impairment could not be totally judged from brain image. Results from our study based on longitudinal, representative population-based design provided support that previous *M*. *pneumoniae* infection is associated with incident speech and language impairment.

Previous studies reported that several factors may be involved with speech and language impairment, including male [24], perinatal factors [23, 24], hearing status [24, 26], medical conditions [30, 31], multiple birth [33, 34], oral sucking habits [35], temperament [36], family history of speech and language problems [37, 38], educational level of mother and father [39], parental mental health [40], maternal age at birth of child [33], birth order [41, 42], and neighborhood disadvantage [42, 43]. Mental handicap [26, 27], autism [25, 26], and attention deficit hyperactivity disorder [29] were also related to speech and language impairment. The findings in this study that boy, preterm labor and small for gestational age, intellectual disability, and other disorders of the CNS were associated with speech and language impairment are comparable with previous studies. But the results of this study that perinatal complications, autistic spectrum disorder, attention deficit hyperactivity disorder, otitis media, hearing loss and traumatic brain injury were not statistically related to speech and language impairment are inconsistent with previous studies. Differences in the size and nature of the samples, the case definition of speech and language impairment and the range and number of possible predictor variables included in the analyses may be the main reasons for the observed differences in study results [24]. Further research is needed to study the effects of factors which are inconsistent with previous studies on speech and language impairment, including perinatal complications, autistic spectrum disorder, otitis media, hearing loss and traumatic brain injury.

Although most of the *M*. *pneumoniae* infection in children younger than 5 years of age were subclinical [10], the influence on speech and language impairment was worthy of attention. In this study, children at the age of *M*. *pneumoniae* infection less than 6 years old have significant risk to have subsequent speech and language impairment.

In most of the *M*. *pneumoniae* infected cases reviewed, respiratory illness are usually mild and has preceded or coincided with the CNS findings [5]. And the development of several neurological findings is indicative of focal or diffuse CNS injury. The definite etiopathogenic mechanism is unknown. Several pathogenic theories have been put forth in recent years, including direct neuroinvasion, neurotoxin elaboration, hypercoagulable state and infection leading to immune dysfunction, such as autoantibodies production [5, 44–46]. It is possible that multiple mechanisms may be occurring simultaneously. For our study, immune complex mediated injury may be one of the pathogenic mechanisms. Through this mechanism, *M*. *pneumoniae* derived antigenic elements may act as molecular mimics and initiate complex immune reactions which subsequently generate neurological injury. A variety of autoantibodies production, such as antibodies against the mitotic spindle apparatus, the centriole, cardiolipin, smooth muscle cells, and lung, liver, and brain antigens (e.g. anti-neuronal antibodies) and IgM-class cold agglutinins have been reported after *M*. *pneumoniae* infection [47–51]. Another possible mechanism is the hypercoagulable state which could result in neurological injury from intravascular coagulation and thromboembolic phenomena of the cerebral vasculature. And immune mediated vascular injury and the development of a vasculitis could further contribute to such effects [52, 53]. It requires further empirical study to explore the putative mechanism of *M*. *pneumoniae* -induced immune response and its influence on speech and language impairment.

### Strength and limitations

The large, nationally representative sample population and longitudinal dataset are key strengths in this study. But there are also several limitations. First, we could not rule out the possible influence of other previously proposed confounding variables not assessed and adjusted for, such as multiple birth, oral sucking habits, temperament, family history of speech and language problems, educational level of mother and father, parental mental health, maternal age at birth of child, birth order, and neighborhood disadvantage. Second, the period of follow-up is insufficient to confirm all possible cases. Speech and language impairment may manifest after the end of the study period in some subjects. Third, the diagnosis of *M*. *pneumoniae* infection and speech and language impairment was established by board physicians and registered in the database. Differences between healthcare providers in their diagnosis of *M*. *pneumoniae* infection and speech and language impairment cannot be controlled for. However, the accuracy of disease diagnoses in national health insurance system could reach up to 85% [54]. Fourth, because of lack of brain imaging data in this study, it could not explore whether the emergence of speech and language impairment was due to brain damage after *M*. *pneumoniae* infection or not.

## Conclusions

*M*. *pneumoniae* infection is temporally associated with incident speech and language impairment. Clinicians should advise parents of children with *M*. *pneumoniae* infection to attend to symptoms that are associated with the development of speech and language impairment, especially when the child is less than 6 years old.
